# Transforming Integrated Care Through Co-production: A Systematic Review Using Meta-ethnography

**DOI:** 10.5334/ijic.7603

**Published:** 2024-03-08

**Authors:** Susan Conquer, Richard Iles, Karen Windle, Rachel Heathershaw, Chantal F. Ski

**Affiliations:** 1 University of Suffolk, UK; 2 Guy’s and St Thomas’NHS Foundation Trust, UK; 3 Queen’s University Belfast, UK

**Keywords:** co-production, co-delivery, patient and public involvement, meta-ethnography, integrated care design and transformation

## Abstract

**Introduction:**

There is a requirement for health and care systems and services to work on an equitable basis with people who use and provide integrated care. In response, co-production has become essential in the design and transformation of services. Globally, an array of approaches have been implemented to achieve this. This unique review explores multi-context and multi-method examples of co-production in integrated care using an exceptional combination of methods.

**Aim:**

To review and synthesise evidence that examines how co-production with service users, unpaid carers and members of staff can affect the design and transformation of integrated care services.

**Methods:**

Systematic review using meta-ethnography with input from a patient and public involvement (PPI) co-production advisory group. Meta-ethnography can generate theories by interpreting patterns between studies set in different contexts. Nine academic and four grey literature databases were searched for publications between 2012–2022. Data were extracted, analysed, translated and interpreted using the seven phases of meta-ethnography and PPI.

**Findings:**

A total of 2,097 studies were identified. 10 met the inclusion criteria. Studies demonstrated a variety of integrated care provisions for diverse populations. Co-production was most successful through person-centred design, innovative planning, and collaboration. Key impacts on service transformation were structural changes, accessibility, and acceptability of service delivery. The methods applied organically drew out new interpretations, namely a novel cyclic framework for application within integrated care.

**Conclusion:**

Effective co-production requires a process with a well-defined focus. Implementing co-delivery, with peer support, facilitates service user involvement to be embedded at a higher level on the ‘ladder of co-production’. An additional step on the ladder is proposed; a cyclic co-delivery framework. This innovative and operational development has potential to enable better-sustained person-centred integrated care services.

## Introduction

The World Health Organisation (WHO) recommended health and care systems partner with service users to create a culture that allows for the co-production of healthcare outcomes [1]. Integrated care services have progressed this recommendation, moving services from working alongside users as passive recipients of care, to active involvement in the development and design of service provision [2]. Recent legislation and guidance around co-production supports this progress, including the European Commission‘s technical dossier for the role of citizens in governance and service delivery [3], the WHO’s principles of integrated care [4] and the Care Act in England [5]. Co-production is acknowledged as an approach where professionals share power and have an equal partnership with people to plan, design and evaluate together [6].

There are many definitions of integrated care [7]. Defined as a global principle to improve patient access and outcomes by organisations delivering services in a joined-up and person-centred way [8], integration can be horizontal (between providers at the same level) or vertical (between providers at different levels), aligning governance structures, budgets and planning approaches [9,10]. The clinical advantages of integrated care have been identified as; better detection of illness, improvement in overall health outcomes and patient experience [11].

Co-production within integrated care recognises that knowledge from service users and providers is essential [12], it supports people with long-term health conditions [13], affects quality improvement [14] and is crucial in mitigating health inequalities [15]. Co-production of change processes generate a wide array of ideas to improve the service user experience across multiple settings [16]. Questions remain regarding the contexts in which co-production is impactful [17,18], as successful outcomes depend on the setting and subjective experiences of those involved [19,20]. While co-production within integrated care is significant to health and care development globally [21], there remains a shortfall of evidence about its impact, with few studies incorporating primary data collection.

Integrated care is too complex for a one-size-fits-all solution [7]. Co-production has the promise of delivering tailored integrated care services [22,23]. For success in a system approach, the needs of all stakeholders including service users and carers must be considered [12,16]. While involving service users throughout a whole-system transformation is a key mechanism for change [24], there is complexity in implementing co-production at a systems level [25,26]. Two key barriers to understanding how to embed co-production within a health and care system include insufficient knowledge of evidence-based methods [23,27], and a lack of preparation to embed into existing structures [18,19,28,29].

**Figure 1:**
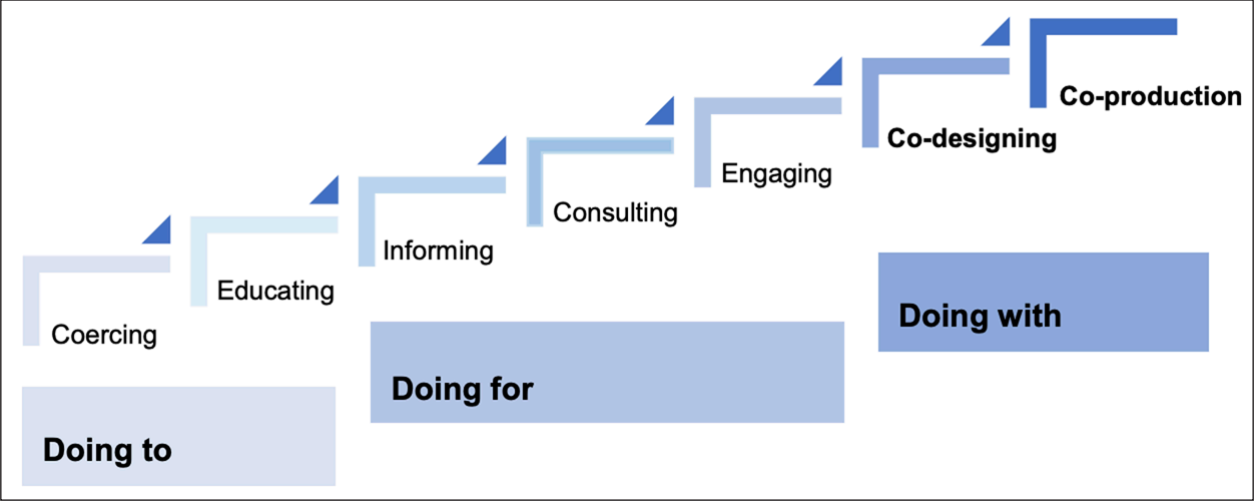
A depiction of the ladder of co-production [35].

‘True’ co-production is part of a continuum of participation, derived from the work of Arnstein [30] and often analogised in literature as a ‘ladder of co-production’ (Figure 1). This conceptualisation interprets co-production as an approach for organisations to share power between citizens, service users, professionals and decision-makers [31,32]. Equalising power through co-production enables service users and carers to act as experts in their conditions and circumstances, and to use this lived experience to develop models of service design and delivery [33,34].

This paper aims to review and synthesise evidence that examines how co-production with service users, unpaid carers and members of staff, can affect the design and transformation of integrated care services.

## Methods

This systematic review was conducted using meta-ethnography together with patient and public involvement (PPI) to explore and synthesise the data. Meta-ethnography is a seven-phase process proposed by Noblit and Hare [36]. It was selected for its ability to add an additional level of interpretation of primary qualitative studies, along with its capability to deliver a comparison of the complexities of co-production across various global contexts [37]. It also enables the full in-depth involvement of a PPI group across the phases; as recommended in eMERGe guidelines for meta-ethnographies [38]. This paper follows the eMERGe reporting guidance [39]: phase 1 is the introduction; phase 2 through phase 6 detail the methods and findings; phase 7 forms the discussion.

**Figure 2:**
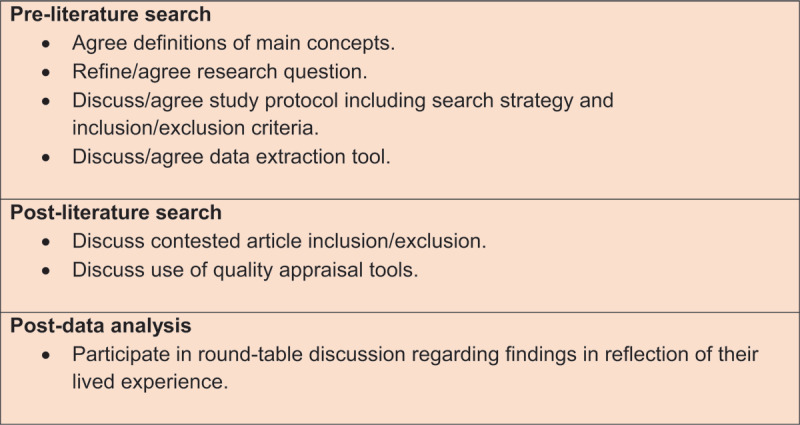
CAG action log.

A Co-production Advisory Group (CAG) of six members was formed comprising service users (n = 2), unpaid carers (n = 2) and members of staff (n = 2) who use and/or provide health and care services within one integrated care system in England. They were recruited through a local Healthwatch network via newsletter and word of mouth. Those interested received an information sheet, and completed a simple application form providing their background and experience of co-production within integrated care. The group met three times with additional email and telephone communications, and were remunerated for their contribution. The lead author facilitated meetings and one co-author (RI) represented a member of staff in the group. Figure 2 depicts key tasks performed by CAG members.

### Search strategy

The SPICES framework was used to develop the research question and guided the search strategy [40] (Table 1).

**Table 1:** SPICES framework and resulting research question.


SPICES HEADINGS	REVIEW CONCEPT

Setting	Health and care services within integrated care

Perspective	Service users, unpaid carers, members of staff

Intervention	Co-production

Comparison	Hypothetically, prior to/without co-production

Evaluation	The design and transformation of service provision

Social science method	Qualitative and mixed method

Research question:	**How has co-production with service users, unpaid carers and members of staff impacted the design and transformation of integrated health and care services?**


Nine academic and four grey literature databases were electronically searched for results published between March 2012 and March 2022, applying the search terms derived from SPICES and guidance from CAG members. These incorporated CINAHL, Cochrane, MEDLINE, Web of Science Core Collection, KCI-Korean, SciELO, Proquest Central, PubMed Central and SAGE Journals, HMIC, NICE, Nuffield Trust and Social Care Online. Search terms included keywords and Boolean operators (‘AND’, ‘OR’) (Appendix A).

Two authors (SC, RI) screened title and abstracts as per exclusion criterion (Appendix B) to exclude off-topic studies applying Covidence software [41]. Where disagreement occurred, consensus was achieved through discussion. Studies were excluded if they did not demonstrate search terms according to CAG agreed conceptual definitions (Appendix C).

Full-text screening involved two stages. First, CAG members each examined two studies against the inclusion criteria. Second, four co-authors (SC, RI, KW, CFS) met to discuss each source in light of CAG members’ views. One author (SC) hand searched reference lists for further studies.

Studies were critically appraised for methodological and conceptual quality [42]. Two tools were tested, the Critical Appraisal Skills Programme [43], and TAPUPAS framework for quality assessment [44]. Three authors (SC, RI, KW) conducted the appraisal and agreed the TAPUPAS framework provided a more accurate test for quality than CASP. Studies scoring <7 points using TAPUPAS were excluded.

### Data extraction

A data extraction tool was designed by co-authors and CAG members. Two co-authors (SC, RI) performed data extraction. Information and direct citations were extracted from each study. The lead author identified emerging concepts from studies and scored the extent of co-production processes through applying the Ladder of Co-production developed by Think Local Act Personal [45] and the Spectrum of Involvement recommended by NHS England and NHS Improvement [46] respectively (Appendix D). Higher scores indicated more active involvement and influence on design and transformation decisions by service users, carers and members of staff in co-production.

### Data analysis

Analysis of concepts of the studies, in order of publication date, was completed by two authors (SC, RI) to determine key themes. Study comparison was separated by aspects of ‘design’ and ‘transformation’. Once broad themes were established across studies, the original texts were analysed by three authors (SC, RI, KW). Findings were visually produced to present to co-authors and CAG members.

### Data translation and synthesis

A 90-minute roundtable discussion with CAG members provided findings in reflection of their lived experience. Two co-authors (SC, RI) presented their early impressions of findings, showing study examples to ensure discussions were rooted in context. Key concepts were identified through the discussion transcript. Concepts were compared with data analysis findings through reciprocal analysis (studies and concepts showing similarities) and refutational analysis (exploration of contradictions). Syntheses of findings were completed through analysis, translation and interpretation of themes. This process involved the lead author presenting themes and translations to the co-authors who shared interpretations of findings and came to a consensus on final interpretations.

## Findings

2,097 papers were identified, of these 1,904 were imported into Covidence which removed 760 duplicates. 191 sources were manually sifted, 243 were full-text-screened, resulting in 21 studies for review and discussion. Ten studies remained, with one additional study meeting inclusion criteria from hand searching. 11 studies were critically appraised for quality; one was excluded (Appendix E).

### Study characteristics

Table 2 displays key characteristics of included studies published between 2015 and 2021, across a range of countries and integrated care provisions. Differences were present in the scale of co-production, ranging from accessing a pathway (e.g. cancer screening) [47] to development of a full integrated care system [48]. Models of co-production included co-design [22,49,50,51,52,53], co-delivery [22,47,52,54,55], peer support [22,47,51,54], and co-production along with a quality improvement (QI) framework [49,50,52,54].

**Table 2:** Included studies.


PRIMARY AUTHOR	PUBLICATION COUNTRY	INTEGRATED CARE PROVISION	CO-PRODUCTION MODEL(S)

Kamvura [55]	Zimbabwe	Depression, diabetes, hypertension	Theory of Change Co-delivery

Bruns [22]	South Africa	Human immunodeficiency virus (HIV)	Co-design Peer support Co-delivery

Sarkadi [52]	Sweden	Childhood neurodevelopmental disorders	Co-design Co-delivery QI

Yadav [53]	Australia (conducted in Nepal)	Chronic obstructive pulmonary disease (COPD)	Co-design

Wolstenholme [51]	England	Hepatitis C virus	Co-design Peer support

O’Donnell [50]	Ireland	Frailty	Co-design QI

Eriksson [47]	Sweden	Cervical cancer screening	Representative co-production Peer support Co-delivery

Lalani [48]	England	Ealy integrated care system	Public committee

Van Deventer [49]	South Africa	Childhood malnutrition	Experienced-based co-design QI

Flora [54]	Canada	Psychiatry	Continuous improvement committees Peer support QICo-delivery


Table 3 (Additional file) shows further study characteristics. Disparity was seen in the temporality of the co-production processes (eight months to three years). The aims of integrated care, co-production, and key outcomes of co-production were extracted for comparison. Most assessed outcomes qualitatively such as systemic changes (e.g. a strategy, intervention or pathway design, relational or capacity building), while some quantitively measured outcomes.

Table 4 (Additional file) re-orders studies from highest to lowest according to their accumulated score using the ladder depictions [45,46]. One study scored the highest (12 points) [47], one the lowest (six points) [48], with the remaining eight studies scoring nine, 10 and 11 points. This order was used to identify any links between the extent of co-production and data extracted.

Findings from the re-order showed two promising links. In exploring the scale of the co-production (size of the health/care provision), one study [48] exploring co-production within an integrated care system scored the lowest, while co-production of access to a cancer screening programme scored the highest [47]. The second link was between the extent of co-production and the model of co-production; the five studies that had a ‘co-delivery’ model [22,47,52,54,55], and the four studies with ‘peer support’ [22,47,51,54] scored within the highest ranked studies.

Table 4 further details numbers of service users, carers and members of staff involved in co-production, demographics, evidence of equity, and evaluation techniques. There was a lack of detailed information across studies which did not support an in-depth understanding of how co-production impacted integrated care design and transformation.

Table 5 (Additional file) shows definitions of co-production which were similar, featuring the bringing together of various actors working collaboratively and equitably using solution-focused approaches. A variety of facilitators, barriers and recommendations are shown. A universal barrier was time and resource constraints challenging the commitment and focus of those involved. Other data includes methods of co-production, who was involved, recruitment strategies, and equalising factors between members.

### Key themes: design and transformation

**Figure 3:**
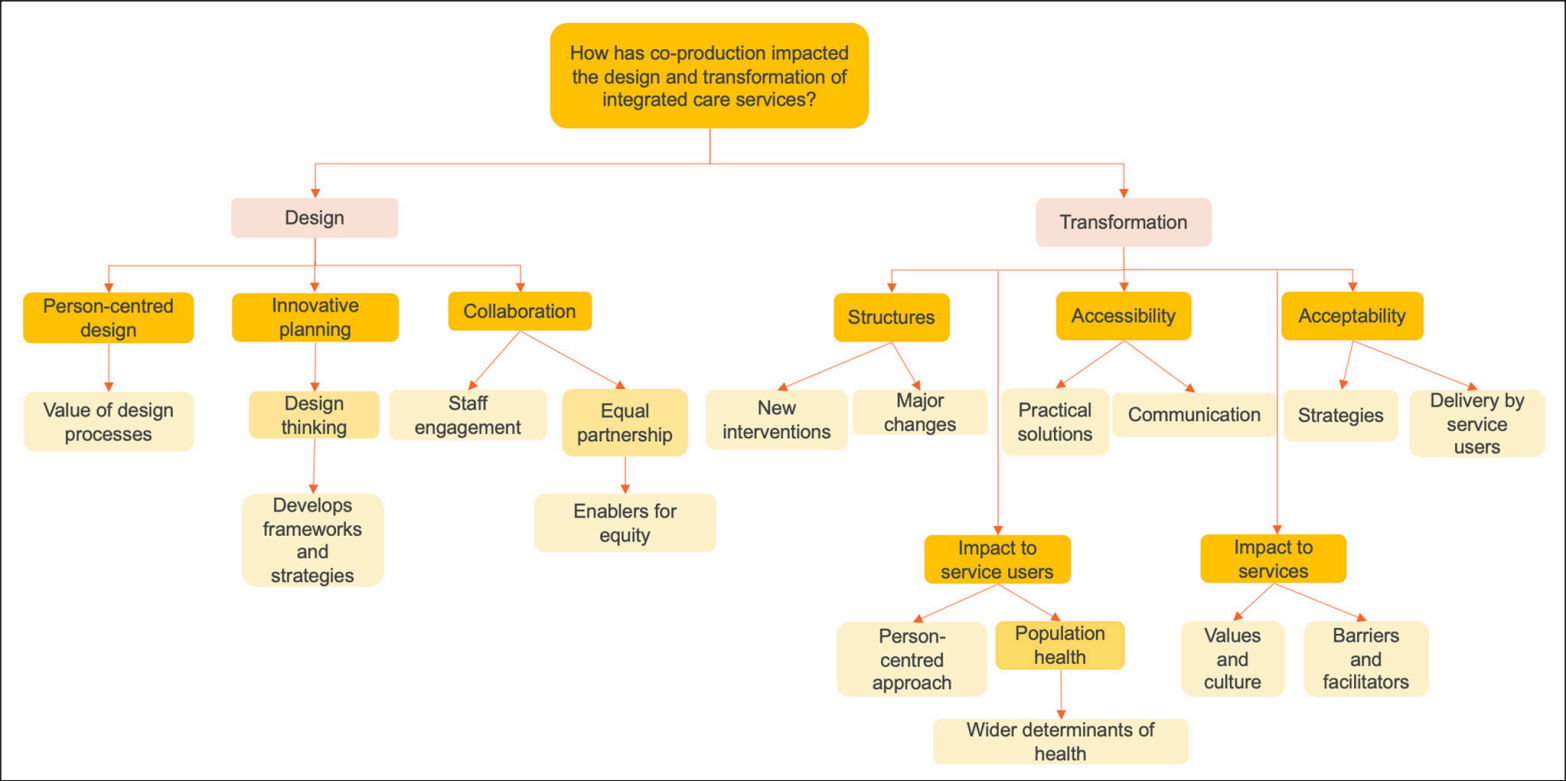
Analysis themes.

Thematic analysis resulted in emergence of two categories: ‘design’ and ‘transformation’ (Figure 3). Design related to the act of developing new service processes in co-production with service users, carers and members of staff, whilst transformation related to implementation and outcome of co-produced changes.

#### Design

Three impacts of co-production on design of integrated care services were identified; person-centred design, innovative planning and collaboration. Challenges and requirements for co-producing design were also drawn from the studies.

##### Person-centred design

Most studies explicitly discussed the importance of service users bringing expertise from their experience in accessing and using services [22,47,48,52,53,55].

“The […] design approach ensured empathy in understanding how men living with HIV in South Africa experience the world […]. This depth of empathy also empowered decision-makers [to] achieve better outcomes and [it] allowed for robust, rapid evaluations of prototypes.” [22: 241]

Person-centred design was described as a vital ingredient [22], with service user input seen as a resource [49], and, as a strategy to reduce health inequalities [47].

##### Innovative planning

Co-production supported development and use of strategies and frameworks within service design. These delivered sustainability of design as well as ensuring co-production processes could be embedded and flexible [50,54,55].

“As phased implementation of the pathways is ongoing, the service and quality improvement initiatives […] are being evaluated using a Plan Do Study Act (PDSA) process. This will generate an iterative process […] whereby the co-designed models of care are continually adapted to the context […].” [50: 4]

Strategies included frameworks of design e.g. journey mapping [22,47,53], developing personas [22,51], design thinking techniques, i.e. democratic dialogue [50], and imaginative questioning [47]. Each approach shaped the process of co-production, supporting accessibility [51], inclusion [55], and collaboration between different groups [49].

##### Collaboration

Collaboration within co-production allowed equity between stakeholders at varying levels and roles within integrated care [22,49,50,54]; studies highlighted enablers for equality [47,50,53,54]. For example, clinical staff rotated their involvement to ensure a higher proportion of public and patient representatives at meetings, to amplify their voice and input [50]. Co-production impacted engagement of staff members and supported collaboration [47,49,52,53]. In one study [49] staff were emotional regarding motives for working in healthcare, their organisation was humbled by how staff discussed service improvements.

##### Challenges

Challenges of co-producing service design were highlighted through learning and requirements [47,48,49,51,52]. The independent evaluation of an early integrated care system [48], was the only study to face a disproportionate number of barriers to facilitators. Co-production was intended across the whole system, but only implemented at smaller scales. This study showed:

1. Co-production could be undermined within financial restraints.
2. Health professionals struggled to work in partnership with service users and felt threatened by their active involvement.
3. A lack of awareness about co-production and how to usefully involve service users.

Importance of tackling barriers with communication and honesty was demonstrated.

“[…] residents were often willing to accept […] tight financial parameters […] but communication has to be effective and residents need to feel that they are being engaged and involved in decision-making.” [48: 34]

“I think one of the problems is we are not very good at being totally honest about things […]. What we probably don’t say to people often enough is, ‘How do you want us to use this money, because that’s all we’ve got?” [48: 34]

##### Requirements

Requirements for successful co-production within service design were stated across the studies, including:

1. Offered information in accessible languages and formats explaining context, encouraging participation [47,48,49,50,51,53].
2. A process wherein service users and carers felt that they were engaged and truly involved in decision-making [22,51,54].
3. Allowed time to build relationships with each participant, involving them in creative processes, to understand the community [51,53,54,55].

#### Transformation

In exploring the impact of co-production on transformation, three key impacts were detailed; structure, accessibility and acceptability. In addition, two further impacts were highlighted, the health of service users and the culture of services.

##### Structure

The use of co-production resulted in substantial physical changes to provision and delivery of services [22,49,50,53,55], e.g. a children’s ward moved floors in a hospital building to integrate paediatric outpatients with an HIV clinic [49]. Structural changes allowed for capacity building [50,53,54,55], a top-down, bottom-up approach [50,52,53,55] and sustainability [22,52], recognising co-production as crucial in allocating resources and ensuring ownership of designed solutions.

Co-production enabled design of new interventions across all studies, e.g. a frailty screening tool [50], and a telephone reminder service [51] were implemented. One study stated the breadth of co-produced interventions [49].

“…a total of 38 concrete, practical QI interventions were suggested […]. Of these, 25 were implemented […] and 5 others were being discussed.” [49: 10]

##### Accessibility

Co-production increased accessibility of service provision through improved communication and pragmatic solutions. Service-to-service user communication supported accessibility [47,50,51,52,55] as service users became more informed about how and why a service met their needs, e.g. through co-produced leaflets [50], posters [51], films [47,51], online platforms [52,55], and radio [47]. Pragmatic solutions to accessibility included a mobile Hepatitis C clinic van [51].

##### Acceptability

Co-production enhanced acceptability of transformations by enabling specific suggestions of strategies [22,51,54,55], e.g. an incentive scheme evaluated over three months demonstrated feasibility and improved attendance rates [51]. Studies showed service delivery being performed by service user representatives [22,47,52,54,55] and each study that featured co-delivery found ownership of the co-produced transformation together with acceptance of change. One study [22] demonstrated the key benefits of the Coach Mpilo programme where men with HIV were recruited as coaches. The unique value of the coach role was trust. Peer support was utilised within the studies, alongside co-delivery [22,47,51,54], as well as people with lived experience providing training [22,52,55]. In one study a peer coordinator was employed [51].

“In supporting future parents, they offered information in their mother tongues, helped to explain the Swedish healthcare system, and participated in parental education together with staff at the local antenatal clinics.” [47: 300]

#### Impact on service users and services

Structure, accessibility and acceptability also affected service culture and service user health outcomes. Co-production has supported services to be more person-centred [22,50,51,52,53,54], e.g. service users and carers emphasised the importance of continence care in quality person-centred care, particularly around dignity and respect, and was prioritised as a pilot [50].

Three stand-out examples of co-production affecting people’s health outcomes were:

1. 42% increase in cancer screening annually [47].
2. Increase in patients getting out of bed and dressed to reduce risks following hospital admission [50].
3. Reduction in death rates from malnutrition; zero over three months [49].

Wider determinants of health were affected by co-production including community-capacity building, social isolation, education and a focus on equality and diversity (e.g. studies featuring co-delivery increased employment and volunteering).

Each study outlined the impact of service culture and values within integrated care, placing service users at the forefront of responses to required transformations. Two studies acknowledged that co-production is a response to a hierarchical system [49,54].

“The [project] acknowledge[d] a paternalistic system and the potential to change this by co-creating solutions, innovations with practical outcomes that were a synergy between previously disparate groups (providers and clients).” [49: 11]

To deliver co-production, each study demonstrated the necessity to put in place a detailed focus, evaluation, reflective and iterative practice. Those featuring QI methods [49,50,52,54] emphasised the importance of testing those developing ideas that come from co-production.

“The project had to be responsive to the emergent ideas and so a range of methods to ‘evaluate’ and test these ideas were developed in collaboration with the project team and workshop participants.” [51: 218]

Each study outlined that co-production could lead to short-term challenges in delivering re-designed services. Examples include:

1. Overburden; too many referrals so community workers struggled with workload capacity [55].
2. Lack of financial resources to implement well-planned structural suggestions [49,51].
3. Slower pace; flexibility required for patients of psychiatry continuous improvement cycles [54].

### Translation and interpretation of themes

The categories ‘design’ and ‘transformation’ also emerged during translation. CAG members affirmed study findings were valid, meaningful and sensible in comparison to their experience of co-production within integrated care. While the themes drawn from the papers were well known, the examples and context provided new knowledge, enabling novel discussion. Reciprocal and refutational analysis were both useful during translation.

**Figure 4:**
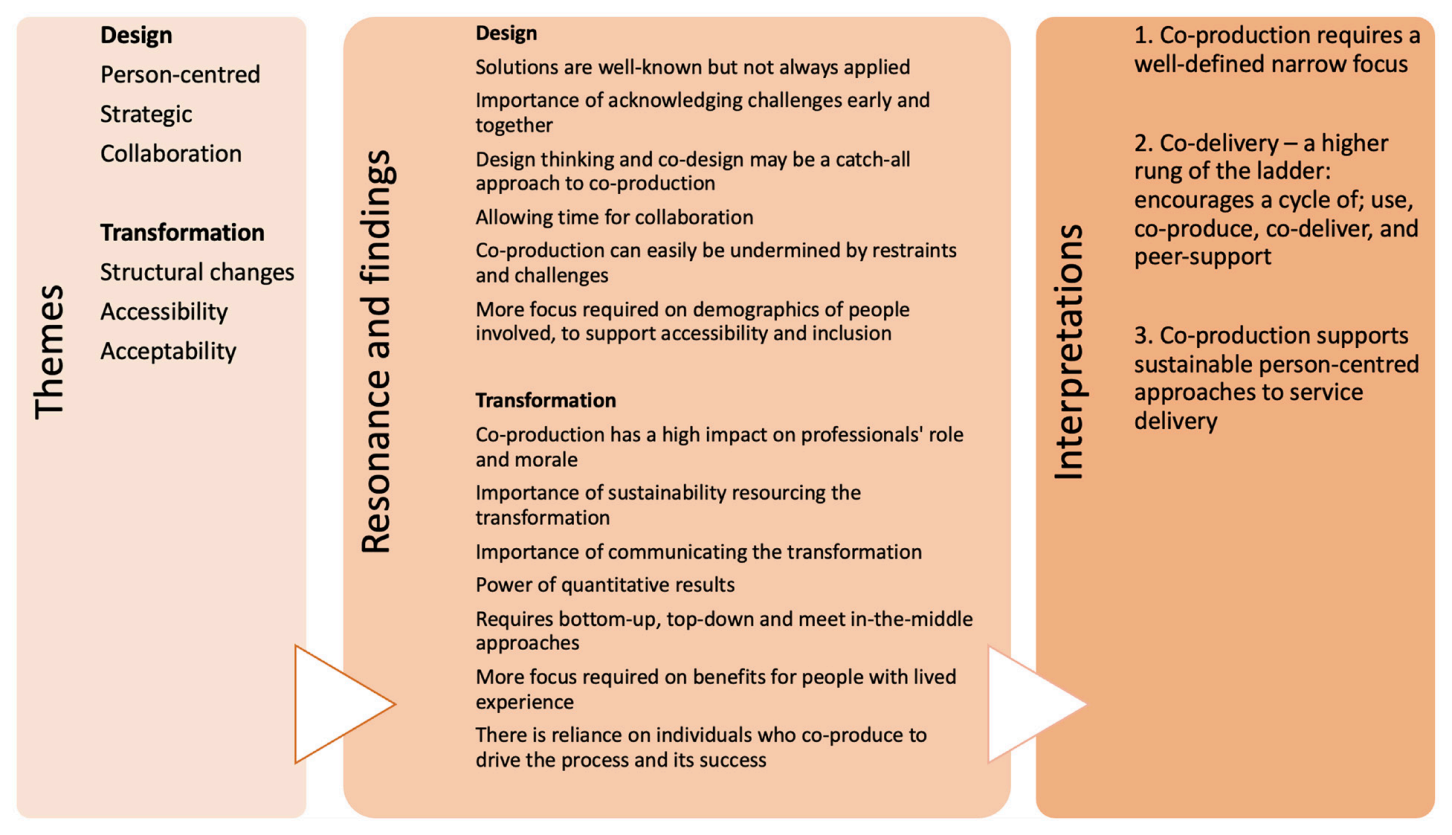
Translation of themes by the CAG into new interpretations.

The CAG recognised the barriers to co-production. The study set within an integrated care system in England [48], which faced the most challenges, became a comparator to the more successful co-production examples. This comparison highlighted the limitations and successes of co-production within both design and transformation. The group’s resonance with the findings were translated into statements which encompass facilitators and barriers. This process provided three new interpretations of the data (Figure 4).

### Narrow focus

Co-production within integrated care is most constructive when matched with an explicit focus. In the case of the integrated care system, the elements of proven co-production were specific projects. Focused co-production processes require a defined scope, large or small, where roles of people involved are clear and vision and scope are shared. Delivery of focused well-defined co-production within integrated care may also require a project leader(s) who supports planning, management and definition of the process including scope, resources available, accessibility, evaluation and reporting outcomes. The CAG and co-author discussions explored the narrow focus required at the design stage, necessity of acknowledging challenges of co-production early and together, importance of co-design frameworks, and need for time to collaborate.

### Co-delivery

The synthesis indicates that co-production benefits from an additional step, that of co-delivery. Building upon the depictions of the ‘ladder of co-production’ [35,45], this review offers evidence for an additional rung with ‘co-delivery’ following ‘co-production’. Co-delivery is where service users and carers who have been involved in co-production then take action to provide the service to others, supporting the operation of the transformation. Demonstrated benefits of co-delivery included ownership of the transformation, individual personal development, acceptance of co-produced changes, and public knowledge about health and care. Studies that co-delivered service transformation also showed the most authentic or ‘true’ co-production approach.

**Figure 5:**
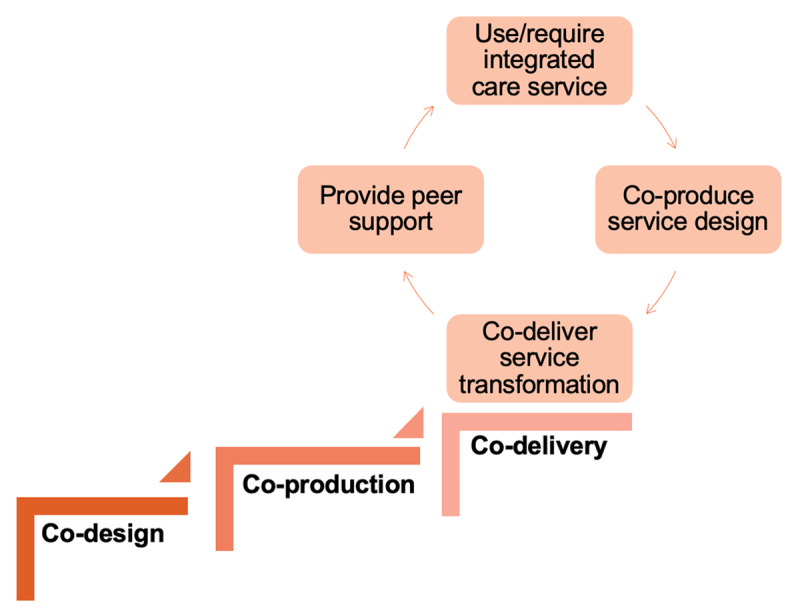
The co-delivery cycle.

The potential of co-delivery opens up a cyclic journey for service users and carers, moving from being the recipient of an integrated care service, to co-production using their lived experience of that service and/or condition, to co-delivery, and finally, peer support (Figure 5). Peer support ensures that future service users can access services, leading to co-production opportunities. This may provide a sustainable implementation process, including co-delivered training.

CAG members and co-authors explored co-delivery with peer support within both design and transformation, in particular, increased acceptability of service changes. Where user involvement is extended to allow individuals to become part of delivery, the impacts are felt on professionals’ roles and morale, longer-lasting involvement benefits for people with lived experience, an increase in resources available in service delivery, and encouraging a bottom-up and top-down approach.

### Sustainable person-centred services

Co-production focuses on the process of designing services so the needs of service users are met early on. Where co-production is well-led, focused and well-defined, co-delivered with people who have lived experience and that process sustains itself through peer support, the processes of co-production can be embedded into the culture of organisations, increasing the possibility that transformations will be person-centred. Where organisations expend their resources to co-produce change, and this has been successful, direct positive outcomes are shown for service users, personalised services, accessibility, and the potential for sustainability. This core concept drawn from the data resonated with CAG members and co-authors, who explored that co-production, co-delivery and peer support could transform services even during their operation by continuously making adaptations and improvements throughout the co-delivery cycle. In addition, it was recognised that sustainable and person-centred provision demanded communication loops, flexible resourcing and bridging gaps between service users’ and professionals’ lived and learned experience of integrated care.

## Discussion

The relationship between the conceptual literature of integrated care and co-production does not always neatly align. While the dominant integrated care frameworks put citizen and service users’ needs at the centre of the purpose of integration, they are not traditionally included in decisions about how integration affects them and their care [2]. However, health and care systems are encouraged to incorporate users as both recipients and designers of care when planning to integratively design and transform provision [56]. The WHO’s framework for meaningful engagement of people living with non-communicable diseases, and mental health and neurological conditions sets out six key enablers for co-creating healthcare [57]. A number of their recommendations are reflected in this review including; resourcing co-production, practically addressing power imbalances, integrating lived experience into design and delivery processes, building community capacity, and embedding co-production into policy.

The aim of this systematic review has been met by our central finding from this review. We propose an extension of the existing ‘ladder of co-production’, adding a co-delivery cycle that includes peer support; enabling co-production to be embedded within integrated health and care systems.

Co-delivery is prominent in the field of co-production within public services [58,59,60], forming one of the ‘Four Co’s’ presented by Loeffler [61] and described as being more about citizens taking action in service improvement than inputting into co-design meetings. Leoffter [62] describes six types of co-delivery, three being most relevant here; co-implementation of projects; contributing to peer support groups and; co-influencing behaviour change. The cyclic co-delivery framework we propose supports and adds to the existing theories by its potential to sustainably be applied to integrated care, as evidence for co-delivery is less common in the associated health and care literature. The most common practice examples of co-delivery within integrated care are seen within mental health [34,63,64], e.g. the global Recovery College’s formal peer-taught programme [65].

Co-production uses personal strengths, resources and assets from the people involved [12,61,66]. Participatory service design within integrated care similarly demands a variety of skills and consideration of where different people’s strengths lie [16]. Co-delivery with peer support provides service users, carers and staff further opportunities to transform services alongside co-production and co-design, and provides services with access to people’s strengths, resources and assets in different and prolonged ways. Thus, a ‘co-delivery cycle’ becomes a logical extension of commonly accepted interpretations of co-production and co-design within integrated health and care systems. More research on the benefits and risks for those involved in co-delivery and peer support is required for assurance of safety, both for those delivering and for those in receipt of co-delivered services [64].

Successful co-production and co-delivery enable sustainable person-centred integrated care services. Providing care that is both integrated and person-centred is a challenge that participatory approaches may help to overcome [15,67]. However much of the evidence about service user and carer involvement and person-centred services is around the micro level, the personalisation of care, rather than co-production at the design or strategic, meso and macro levels [27,68]. The same can be said for integrated care theories, such as the House of Care approach [69] and The Burrtzorg model [70]. This review included studies at the meso and macro levels. Further research could be undertaken to understand how to embed co-production into existing frameworks at these levels, for example, the Rainbow Model of Integrated Care, which identifies six dimensions of integration aligned with micro, meso and macro levels [71].

The ambition, that co-production creates equal partnerships with people who understand first-hand the needs of the population [13,14], provides a good basis for co-production enabling person-centred integrated care. Co-produced changes, even small tweaks, have been found to improve accessibility and engagement with healthcare interventions [16]. Co-production with a narrow focus within integrated care is seen in literature and the practice of QI processes [49,50,52,54,72,73]. These well-defined methodologies have increasingly used service user involvement [26,74]. Experience-based co-design (EBCD), originally developed by Bate and Robert [75], is a published method of combining QI and co-production to ensure a focused time-bounded approach [76]. While this review initially captured several papers using variations of EBCD, with differing levels of service user and carer involvement, only one study featuring EBCD was included, as the rest did not constitute ‘co-production’ within the agreed definition.

Co-production in practice is often a creative, participatory space where various actors bring ideas for service improvement [47,66,77]. However, creativity may be restricted if the co-production process is limited to a narrow focus, as suggested in the findings. As seen across many of the studies, people involved suggested changes that were not implemented, e.g. a Hepatitis C outreach van was fully co-designed but limitations negated implementation [51]. It can be disappointing for those involved in co-production when the process is focused on the design and transformation without ongoing regard for the realities of resourcing and pace of cultural change. Simultaneously, if co-production is given a too narrow focus the features often desired in co-production (e.g. innovation, trust, equal partnerships) may be lost.

“Co-production is a slippery concept and if it is not clearly defined, there is a danger its meaning is diluted and its potential to transform services is reduced. At the same time, a definition that is too narrow can stifle creativity and decrease innovation.” [Social Care Institute for Excellence, 2013, cited in 51: 213]

Iterative and flexible design approaches, seen in included studies, allow services to be quickly accepted and meet people’s multiple care needs. This review sits in the paradigm of progression in organisational culture and values within integrated care, historically expressed as ‘nothing about us without us’ [22,78]. This ethos puts service users at the forefront of a required transformation, and thus co-production may be a challenge to a paternalistic system [49,54], system-focused frameworks and one-size-fits-all approaches to integrated care service design and transformation.

### Strengths and limitations

The included studies showed strength in their deep descriptions of co-production, clear context, and recommendations for future practice. Meta-ethnography proved to be a valuable iterative process for organically exploring co-production within integrated care design and transformation. Findings and interpretations were drawn from the data using the seven phases. PPI was vital to the protocol development and understanding of the findings, which is highly recommended to future meta-ethnographers. The CAG members’ resonance with the findings provided a rich contribution, enabling new interpretations of the data to be understood at an operational level. The strength of methods resulted in a novel cyclic co-delivery framework for application within integrated care.

The main limitation was gaps in data including; numbers of people within different groups, demographics, evaluation techniques and equality across and between groups (Table 4). It was therefore difficult to compare the models of co-production. CAG members emphasised this lack of data proved challenging for assuring equity across co-production processes, limiting knowledge of accessibility and inclusion of diverse people or communities and learning for future best-practice. They also noted that studies displaying quantitative data could more easily show successes and tangible outcomes than those not providing statistical information via measuring impacts or undertaking an evaluation.

There were limitations to the methods, most notably how depictions of the ‘ladder of co-production’ were used to measure the extent of co-production. As multiple studies scored the same, further work may be necessary to capture nuances of co-production processes. Similarly, heterogeneity of studies in practice and provision limited the application of CAG-developed concepts and definitions relating to ‘co-production’ and ‘integrated care’. This heterogeneity made describing each model of co-production challenging for authors and CAG members. The models were revealed during data extraction therefore not previously defined for inclusion in the search criteria. Despite this, varying concept definitions present in the papers (Table 5), along with use of the scoring process, enabled authors and CAG members to draw conclusions from the data.

## Conclusion

This paper highlights three core findings; co-production requires a process with a narrow focus, co-delivery with peer support facilitates service user involvement to be embedded at a higher level on the ‘ladder of co-production’, and implementing these enables transformations to be person-centred. Through novel use of meta-ethnography and PPI, this review proposes a cyclic co-delivery framework. This innovative and operational development has potential to enable better-sustained person-centred integrated care services.

Further research is recommended to explore how co-production within integrated care, and the cyclic co-delivery framework, can deliver successful design and transformation resulting in person-centred care. In particular, primary data collection and evaluation of co-production methods within integrated care service design and subsequent service transformation need to be prioritised.

## Additional Files

The additional files for this article can be found as follows:

10.5334/ijic.7603.s1 Appendices. Appendix A to E.

10.5334/ijic.7603.s2 Data tables. Tables 3 to 5.
