# Supplementary material for: Transforming Integrated Care Through Co-production: A Systematic Review Using Meta-ethnography
Source: Int J Integr Care. 2024 Mar 8;24(1):17. doi: 10.5334/ijic.7603 (PMC10921964; doi:10.5334/ijic.7603)

Appendix A. Search strategy

Database	Order exported to Covidence	Boolean search terms	Number sources	Number duplicates identified	
PubMed Central	1	“A”[ab] AND “B”[ab] AND “C”[ab]	157	0	
Proquest Central	2	“A”[ab] AND “B”[ab] AND “C”[ab] AND “D”[ab]	350	94	
Web of Science Core Collection	3	“A”[ab] AND “B”[ab] AND “C”[t] AND “D”[t]	351	213	
KCI-Korean	4	“A”[ab] AND “B”[ab] AND “C”[t] AND “D”[t]	1	1	
MEDLINE	5	“A”[ab] AND “B”[ab] AND “C”[t] AND “D”[t]	311	272	
SciELO	6	“A”[ab] AND “B”[ab] AND “C”[t] AND “D”[t]	2	2	
Cochrane (reviews and trials)	7	“A”[at] AND “B”[at]	169	22	
CINAHL	8	“A”[ab] AND “B”[ab] AND “C”[ab]	299	144	
HMIC	9	“A”[af] AND “B”[af]	41	3	
SAGE Journals	10	“A”[kw] AND “B”[a] AND “C”[a] AND “D”[a]	223	9	
NICE	N/A	“A”[a] AND “B”[a] AND “C”[a]	10	0	
Nuffield Trust	N/A	“A”[a]	104	0	
Social Care Online	N/A	“A”[af]	77	0	
Reference lists	N/A	N/A	2	0	
**Total sources**			2097	760	**1337**

Key

A - “co-production” OR “coproduction” OR “co-creation” OR “cocreation” OR “co-design” OR “codesign” OR “user participation” OR “user involvement” OR “patient participation” OR “patient involvement” OR “patient and public involvement” OR “PPI”

B - “integrated care system” OR “ICS” OR “integrated care” OR “integration” OR “multidisciplinary team” OR “MDT”

C - “design*” OR “transform*” OR “improv*” OR "innovat*" OR “change” OR “evolve”

D - "health*" OR "social care" OR "care*"

ab – abstract

t – topic

at – all text

af – all fields

a – anywhere

Appendix B. Inclusion and exclusion criteria

Inclusion criteria:<br>• Qualitative and mixed-method studies<br>• Published March 2012 – March 2022<br>• Refers to ‘co-production’, ‘integrated care’, ‘design’ and ‘transformation’<br>• Refers to co-production for the benefit of a wider population<br>• Refers to co-production being translated into integrated care practice
Exclusion criteria:<br>• Conceptual or discussion paper<br>• Minor contribution to integrated care<br>• Research PPI not translated into practice<br>• Involvement in the personalisation of care i.e. their care plan

Appendix C. CAG concept definitions

Concept	Definition as agreed with the Co-production Advisory Group
**Co-production**	A process whereby people, including service users and/or unpaid carers, become actively involved and influence the decisions that design and transform health and care services.
**People**	Stakeholders (citizens, service users, patients, unpaid carers or representatives of organisations) who are part of, or impacted in any way by, the decisions made.
**Integrated care systems**	Defined and coordinated networks of partnerships and associations between organisations providing services that meet people's health and care needs across a geographical area. Adapted from (1)
**Service user**<br>**Unpaid carer**<br><br>**Member of staff**	A person who is, or has experience of, using and/or requiring access to health and/or care services.<br><br>A person, usually a family member or friend, who takes a role in the care of a service user without payment for their time and expertise.<br>A person who has a paid or voluntary job role, representing any organisation within an integrated care system, to provide health and/or care services.
**Health and care services**<br><br>**Integrated care**	Provisions available to service users provided or commissioned by statutory organisations, charity and other third sector providers.<br>Health provides clinical services, whilst care provides longer-term, preventative and educational services, supportive of independence. Some services provide aspects of both. Adapted from (2)<br><br>When health and care services work together to coordinate and collaborate for service users to receive more efficient and effective provision for their health and care needs. This may take place between providers operating at the same level (horizontal integration) and between providers at different levels (vertical integration). Adapted from (3, 4)
**Design**<br><br>**Transformation**<br><br>**Design and transformation**	The act of developing new service processes within an integrated care system.<br><br>The implementation and outcome of that change.<br><br>Service design can contribute toward transformation by providing a person-centred, holistic and iterative approach to the creation of new services. Service users, unpaid carers and members of staff are actively engaged in transformation processes through participatory design approaches. Adapted from (5)

1. NHS England. What is Integrated Care? 2021. https://www.england.nhs.uk/integratedcare/what-is-integrated-care/
2. Leutz WN. Five Laws for Integrating Medical and Social Services: Lessons from the United States and the United Kingdom. Milbank Q. 1999 Mar; 77(1): 77-110. https://doi.org/10.1111/1468-0009.00125
3. Kodner DL, Spreeuwenberg C. Integrated care: meaning, logic, applications, and implications – a discussion paper. Int J Integr Care. 2002 Nov 14; 2(4). DOI: https://doi.org/ 10.1111/1468-0009.00125
4. Ham C, Curry N. Integrated care: What is it? Does it work? What does it mean for the NHS? The King's Fund: London. 2011. https://www.kingsfund.org.uk/sites/default/files/field/field_publication_file/integrated-care-summary-chris-ham-sep11.pdf
5. Patrício L, Sangiorgi D, Mahr D, Čaić M, Kalantari S, Sundar S. Leveraging service design for healthcare transformation: toward people-centered, integrated, and technology-enabled healthcare systems. J. Serv. Manag. 2020 Jun; 31(5): 889-909. https://doi.org/10.1108/JOSM-11-2019-0332

Appendix D. The two ladder depictions used within this review

Left: The Ladder of Co-production [45]
Right: The Spectrum of Involvement [46]

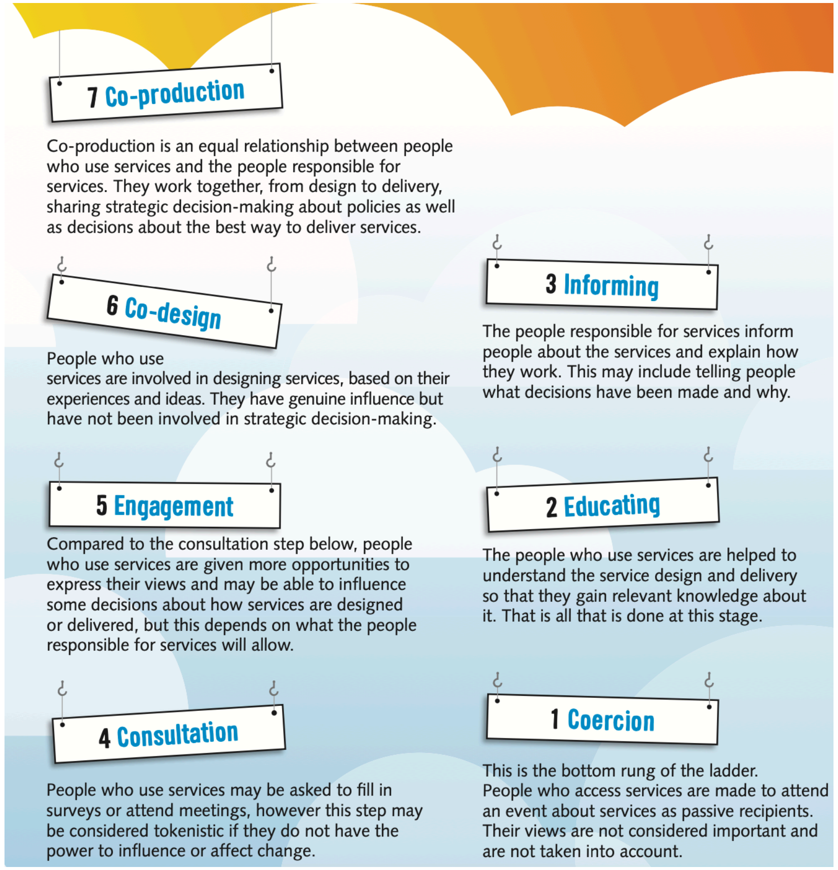

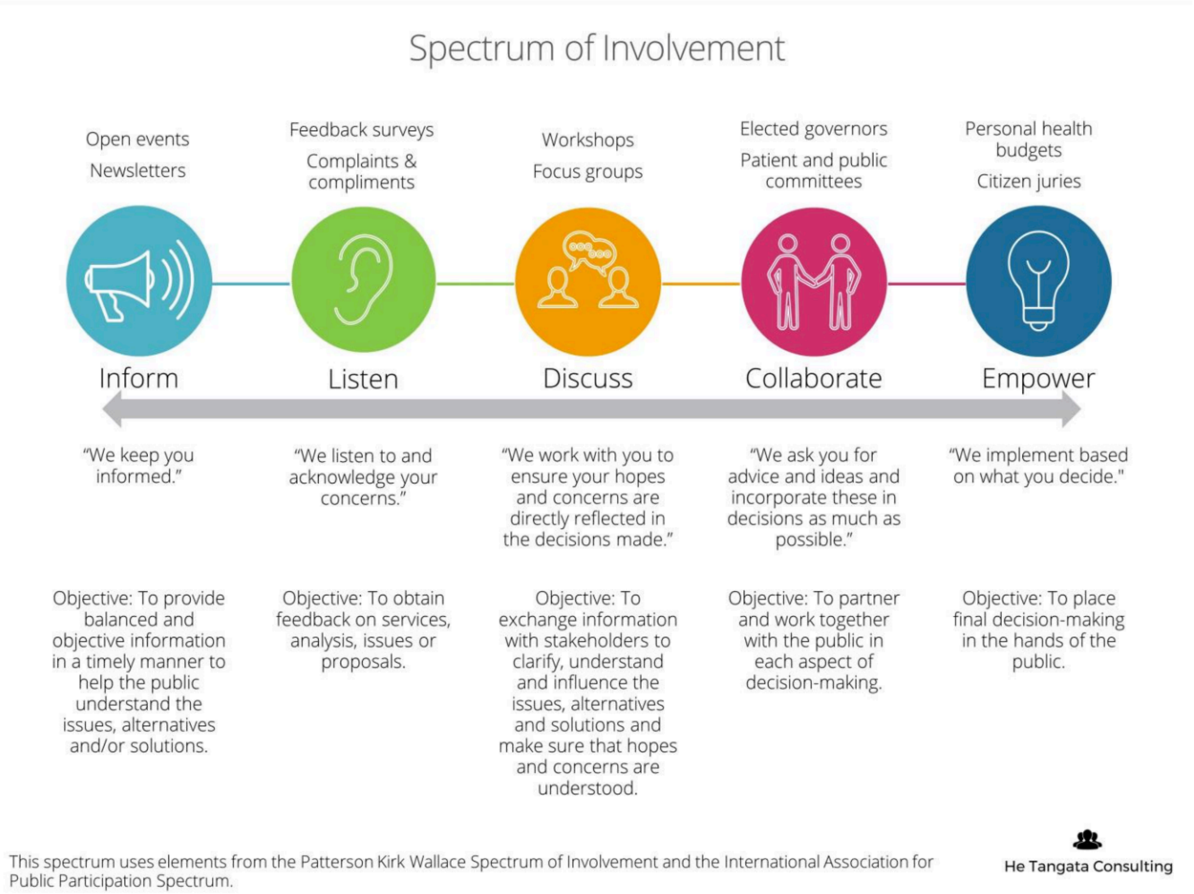

Appendix E. PRISMA flow diagram

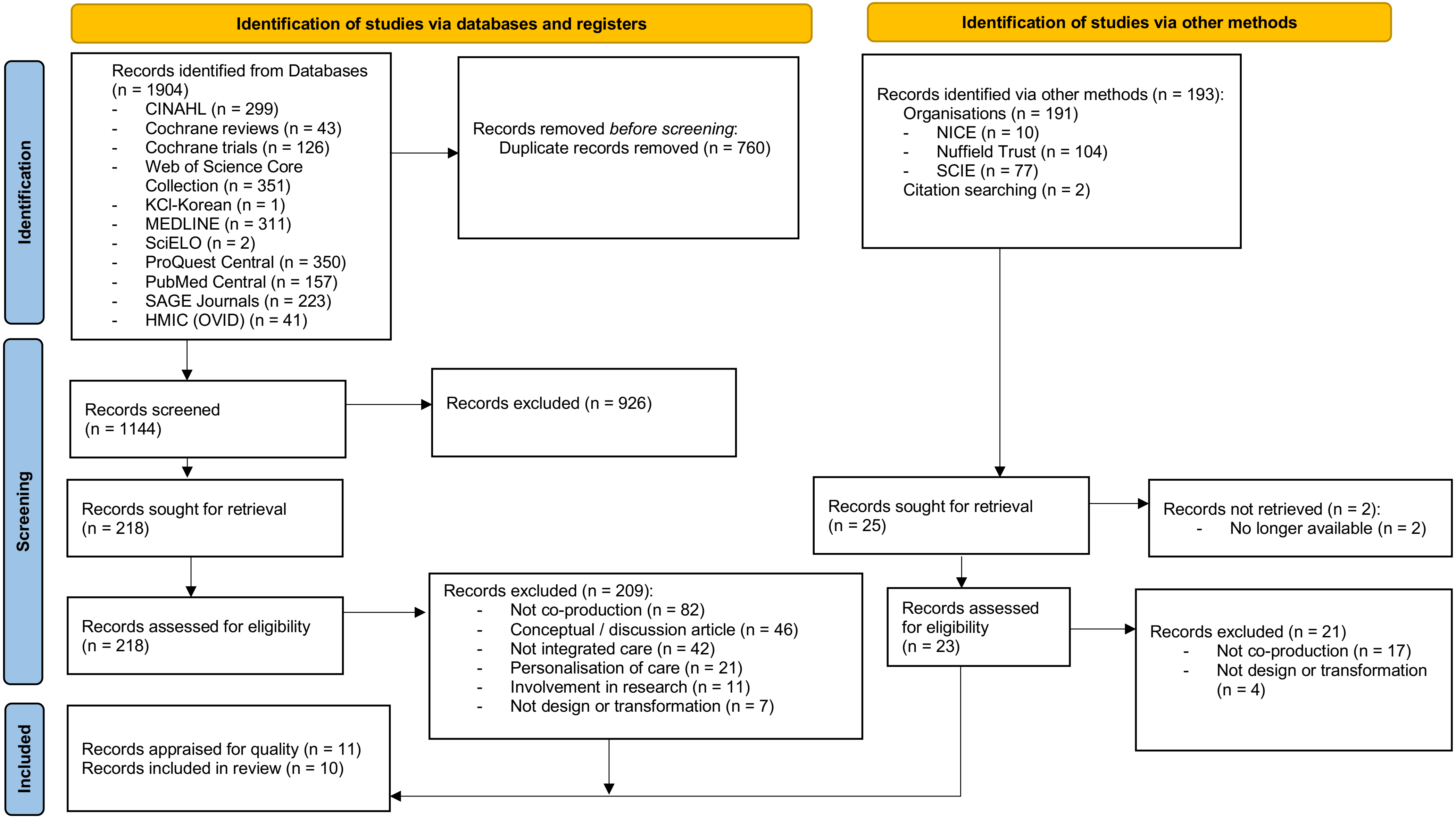

Supplement: Appendices. — Appendix A to E. [file ijic-24-1-7603-s1.pdf]
