# Supplementary material for: Transforming Integrated Care Through Co-production: A Systematic Review Using Meta-ethnography
Source: Int J Integr Care. 2024 Mar 8;24(1):17. doi: 10.5334/ijic.7603 (PMC10921964; doi:10.5334/ijic.7603)
Supplement: Data tables. — Tables 3 to 5. [file ijic-24-1-7603-s2.pdf]

Table 3. Key characteristics of included studies

Primary author name	Year published	Publication country	Health / care provision	Part of provision co-produced	Co-production model	Key aims of integrated care	Aims of co-production	Key outcomes of the co-production	Length of process *estimation
Kamvura	2021	Zimbabwe	Depression, diabetes and hypertension	Pathway and care model	Theory of Change and co-delivery	To integrate depression, diabetes, and hypertension care in primary health care systems	To build consensus and define the pathway to integrate provision into the Friendship Bench care package	A public engagement strategy, leveraging Friendship Bench existing resources, use of "expert clients", inclusion of these local and experienced grandmothers and people with lived experience to co-create the intervention	1 year*
Bruns	2021	South Africa	Human immunodeficiency virus (HIV)	Intervention	Co-design, peer support, and co-delivery	To implement a scalable intervention to increase uptake of HIV testing, prevention, and links to care and treatment	Using a design thinking approach for developing prototype interventions, the selection and pilot testing Coach Mpilo, a peer-support intervention for young men	Workshops >20 solution concepts on four broad themes revealing four different needs, implemented in five areas, employed 120 coaches, and reached over 3,700 men living with HIV, high level of acceptability from and funding partners	3 years
Sarkadi	2021	Sweden	Childhood neurodevelopmental disorders	Care model	Co-design, co-delivery and quality improvement (QI)	To develop a vertical-, clinical- and service-integrated care model to embed a diagnostic process	Involve parents in all steps of the process, from developing and designing the care model to continuously improving it	Parent satisfaction was high, the use of clinician resources efficient and the evaluation period less care than six weeks, a Family Advisory Council was established	2 years
Yadav	2021	Australia (conducted in Nepal)	Chronic obstructive pulmonary disease (COPD)	Intervention	Co-design	To develop an integrated biomedical and psycho-social model of care for self-management support for people with multi-morbid COPD	Co-design more context-specific models for delivering care to deliver a comprehensive intervention in a rural district of Nepal	Five main changes to the prototype following the workshop, a co-designed model of care, building capacity of health facilities and end-users	2 years
Wolstenholme	2020	England	Hepatitis C virus	Access and intervention	Co-design and peer support	A hospital Hepatitis C clinic integrating accessible screening, care, and treatment	To devise solutions to improve access to the nurse-led hepatitis C clinic	Identified four interventions, two were incentives (rewards and enablers) and two were information (peer support and visual communication), engaged a group of service users who are typically hard to access to participate in a project	Not stated
O'Donnell	2019	Ireland	Frailty	Pathway	Co-design and QI	To improve service delivery in the integrated care pathway in an acute hospital setting for frail older people	Consensus building workshops intended to ensure the resulting pathway was patient-centred and responsive to identified and prioritised service delivery need	A number of practical outcomes implemented around key patient-centred outcomes - improved incontinence care, mobility, access to food and hydration, information and signage, opening channels of communication and improving relationships and trust	18 months
Eriksson	2019	Sweden	Cervical cancer screening	Access	Representative co-production, peer support and co-delivery	To increase understanding and uptake of a cervical cancer screening programme in the community, health education and antenatal clinic	To understand immigrant women's experiences, design a context-specific screening programme and increase screening uptake	The information and dissemination methods were improved. Individuals represented their community to deliver the programme. Screenings increased by 42 per cent in a year	1 year
Lalani	2019	England	Early integrated care system	Partnership	Public committee	An integrated care partnership of system and community health services	The role of service users and residents in the design or redesign and development of health and social care services	A platform in which resident's views could be heard with regular attendance from middle managers to discuss service planning and transformation, the committee contributed to the bid which may have been a contributory factor in being awarded the contract, co-design of a health education resource	3 years
Van Deventer	2016	South Africa	Childhood malnutrition	QI	Experienced-based co-design (EBCD) and QI	To address poor nutritional outcomes in a hospital setting	Incorporating EBCD into the QI intervention to enhance childhood nutrition and wellness by increasing patient inclusivity in the ongoing improvement work	38 concrete, practical QI interventions were suggested, 25 were implemented, the most recent malnutrition death rate was zero over the last three months, co-design meetings were a vibrant experience of engagement for patients and staff	8 months
Flora	2015	Canada	Psychiatry	Care model and QI	Continuous improvement committees, peer support, QI and co-delivery	To develop and implement a methodology for continuous improvement of patient and interprofessional collaboration in psychiatry	Design specific tools for diagnosis and transformation practices in partnership with patients and their families and involvement in continuous improvement committees	Embedded within four regions, fostering a sense of continuity, security, and partnership, video of a meeting supporting the development of intervention plan, co-constructed an intervention planning tool, high satisfaction and benefits to patients, relatives and others using the programme	1 year

Table 4. Scores and a re-order of included studies to analyse the extent of co-production processes

Primary author name	Ladders of Participation			New order Primary author name	Co-production model	Numbers of service users, carers (SU&C) and members of staff (MoS) involved	Demographics of service users and unpaid carers involved	Evidence of equity between all co-producers including accessibility	Evaluation or measure of design and transformation (D&T) and co-production (C)
	TLAP /7	NHS /5	Total						
Kamvura	6	3	9	Eriksson	Representative co-production, peer support and co-delivery	SU&C - 20<br>MoS – Not stated	Local immigrant women living who speak Swedish, share culture and language with people the system fails to reach.	Paid on an hourly basis by the healthcare provider	D&T – Number of tests taken retrieved monthly during the intervention and compared with the numbers from two years prior<br>C – Not stated
Bruns	6	5	11	Bruns	Co-design, peer support, and co-delivery	SU&C and MoS - 32	Men living with HIV	The workshop brought men, healthcare workers and staff together as equal partners	D&T - Number of coaches and number of men reached counted. Preliminary data indicate that more than 90% of men are linked or returned to care within the first month of support and remain on treatment thereafter<br>C – Not stated
Sarkadi	7	4	11	Sarkadi	Co-design, co-delivery and QI	SU&C - Not stated, 5 parents formed the Family Advisory Council<br>MoS – Not stated	Parents raising children with neurodevelopmental impairment, some had cognitive challenges themselves	Not stated	D&T - Guided by the Institute for Healthcare Improvement framework, results continually fed back as part of the Plan Do Study Act (PDSA) cycle, used benchmarking models of assessments<br>C – The user involvement process was documented
Yadav	6	4	10	Flora	Continuous improvement committees, peer support and co-delivery	SU&C - 2 per committee<br>MoS – 6 per committee	Have suffered from a mental health illness, stable state of health, significant experience of the health care services, interpersonal skills, desire to help people	Part of continuing professional development for professionals involved, patient coach trains and supports patients, supports before and after each meeting	D&T – Questionnaire<br>C – This paper was the evaluation
Wolstenholme	6	3	9	Yadav	Co-design	SU&C - 4 in advisory group<br>MoS - 6 in advisory group<br>68 total participants in workshop	People with COPD and their family members	Workshop and handouts in Nepali language, two interpreters with a health background attended	Not stated
O'Donnell	6	3	9	Kamvura	Theory of Change and co-delivery	SU&C – 4<br>MoS - 10	People living with non-communicable diseases	Training provided within existing community groups	D&T - A determinant and evaluation framework comprised of context, implementation and setting that interact with one another and the intervention at a micro, meso and macro level<br>C – Not stated
Eriksson	7	5	12	Wolstenholme	Co-design and peer support	SU&C – 12<br>MoS - 10	Patients with a history of injecting drug use, some had completed treatment, male and female, all spoke English	The service users given a £20 high street voucher and their travel expenses were reimbursed, used visual materials	D&T - After 3 months the project was evaluated suggesting it was both feasible to run an incentive scheme and acceptable to patients. Despite the small numbers, the change in attendance rates was encouraging<br>C – Not stated
Lalani	4	2	6	O'Donnell	Co-design and QI	SU&C – 10<br>MoS - 8	Older patients with recent experience of acute care, a family carer, patient with complex needs	Consistently greater representation from public/patients than professionals, public/patient acted as a co-chair, capacity building sessions prior to process, meetings conducted in the same accessible location with refreshments, relaxed atmosphere, informal language	D&T - A series of intervention PDSA cycles, formed the final two co-design meetings<br>C - Review of the process
Van Deventer	6	3	9	Van Deventer	Experienced-based co-design (EBCD)	SU&C - 10<br>5 mothers with 3 children at joint event<br>MoS – 14<br>16 at joint event	Mothers/caregivers with a child diagnosed with malnutrition or HIV positive, from the township, English or Afrikaans	At least 1 mother in each co-design team	Not stated
Flora	7	4	11	Lalani	Public committee	Not stated	Local residents	One resident translated for the Bengali parents	D&T - This paper was an evaluation<br>C – Not stated

Note. Studies re-ordered in ascending order of their accumulated scores using the ladders (the higher scores indicate more active involvement and influence by people involved in co-production). Their co-production model, numbers of service users, carers and members of staff, demographics, evidence of equity and evaluation techniques, within this new order.

Table 5. Definitions of co-production, facilitators, barriers and recommendations of included studies

Primary author name	Definition of co-production	Facilitators to co-production	Barriers to co-production	Main recommendations
Kamvura	A structured approach of bringing together different stakeholders to build consensus on a common initiative	Structured approach, strong presence of the community through the support groups	Over-burdening the grandmothers, COVID-19 poses logistical challenges in implementation	Supplementation by a series of qualitative studies, feedback loops that provide an opportunity to adapt the intervention to suit its context
Bruns	A dynamic approach that engages end-users and key stakeholders to develop tailored, usable products, programs, or systems	Empathy in understanding how men living with HIV in South Africa experience the world and keeping them at the centre of the design process, consider issues of desirability, feasibility, and viability from the beginning, co-design as the project philosophy and its application in the development of the intervention	Lack of trust, errors based on top-down assumptions and biases	Applying co-design principles to the implementation and scale-up of the intervention, consider key issues and potential challenges, including program ownership and accountability, rigorous evaluation and long-term sustainability and commitments for funding
Sarkadi	To involve parents in all steps of the process, from developing and designing the care model to continuously improving it	Having parents in the room changed the conversation and ensured focus remained on the needs of children and their families, rather than organisational priorities and loyalties, employed staff were compensated by the organisation, allowed the team 5% of their time to develop the care model during the first year	A lack of mind-shift for healthcare managers, professionals, and users	Having patience with the process, initial investment is required including managing<br>resistance, building relationships across professional boundaries and problem-solving
Yadav	A process of collaborative design thinking: a process of joint inquiry and imagination in which diverse people jointly explore and define a problem and jointly develop and evaluate solutions	Meeting with people with COPD, family members and caregivers, primary health care workers in their own local environment, face-to-face meetings with top-level stakeholders, pre-workshops, frequent visits to the community leaders and patients homes, facilitators to be from the same cultural or geographic background and previous experience working with the local community	The co-design process is time-consuming especially the lead-in time, working around the schedules of the different stakeholders, engaging patients from a marginalised community	Using both top-down and bottom-up approaches to develop an integrated model of care
Wolstenholme	A meaningful engagement of all stakeholders in the design of new services or knowledge	Workshops held in a neutral venue that was easily accessible for participants using public transport, using a creative co-productive approach, warm-up activities, the use of personas and other creative methods	Service users dropped out, not all the stakeholders were able to participate in every workshop due to work commitments.	Nurses should be at the forefront of facilitating the involvement of individuals and their carers in co-designing and providing care services
O'Donnell	An approach within health system improvement initiatives involving creating an equal partnership of people working within the system and those individuals who have lived experience of using the system	Terms of reference were agreed at the first co-design meeting in which participant roles were clarified and defined, financial and administrative supports, securing institutional commitment, respectful and clear communication between healthcare personnel and patients, identifying a diverse range of public and patient representatives through third party organisations.	The necessity to consider local contexts before the adoption of the design approach, requires considerable time and resources from all involved, can be overly representative of public/patients who are engaged and interested in QI and who have the cognitive and socio-economic capacity to engage	The public/patient voices in the co-design process focused the attention of the organisational leadership on the priority areas that were identified
Eriksson	The joint and voluntary involvement of group representatives in evaluating, designing, and delivering public services that enable value co-creation for other group members	The knowledge and skills and networks of the representatives, midwifes acted as a cultural bridge between local women and staff	Representatives are likely to be quite distant from the target group	Involve representatives of groups that the public management knows little about, long-established relationship may bridge the user-provider gap, management and researchers should address not only the core service, but also co-production activities impact on the broader service system
Lalani	To partner with the public in each aspect of the decision including the development of alternatives and the preferred solution	Senior and middle managers champion co-design and co-production, culture shift for health professionals, bottom-up engagement and involvement of individuals at all levels (including service users), a combined vision and sense of ownership, asking 'how' and 'why'	Not being honest about the budget, lack of knowledge about how to do co-production, staff becoming frightened of what to say in front of patients and users	Service user/residents to get involved in key components of a programme cycle, local Health Wellbeing Committees should involve users/residents at an early stage in their development, a system-wide strategy that mandates the involvement of service users/residents where appropriate
Van Deventer	The collaborative planning and implementation of solutions by mothers/caregivers and staff	The attitude of co-operation from most of the hospital management and the enthusiasm of the patients that remained involved, holding a celebratory event for everyone involved, providing a clear ending point to this part of the project	The process of recruiting patients and retaining them, language barriers, time consuming and costly	EBCD is a clearly defined process which allows patient perceptions and emotions to emerge as part of a planned change or improvement process
Flora	It brings together three inseparable perspectives, clinical managers, workers and patients (or relatives caregivers), to integrate and realise common aims of the project together	Working in smaller groups, patients support each other	Patients leaving the process before the end, dependent on the will of all – patients, loved ones, managers and caregivers – to work together to face the major challenges currently facing our health network	All committee members accepting to experience the partnership and by recognising the wealth of knowledge of all
